# Satisfaction with Maternity Care Among Immigrant Women, Refugee Women and Non-immigrant Women: Results of the Pregnancy and Obstetric Care for Refugees (PROREF) Cross-Sectional Study

**DOI:** 10.1007/s40615-025-02529-z

**Published:** 2025-07-02

**Authors:** Vera Seidel, Louise Teschemacher, Jürgen Breckenkamp, Theda Borde, Matthias David, Michael Abou-Dakn, Wolfgang Henrich

**Affiliations:** 1 Klinik Für Geburtsmedizin, Charité Universitätsklinikum, Campus Virchow-Klinikum, Augustenburger Platz 1, 13353 Berlin, Germany; 2 Deutsches Herzzentrum Der Charité, Klinik Für Angeborene Herzfehler Kinderkardiologie, Campus Virchow-Klinikum, Augustenburger Platz 1, 13353 Berlin, Germany; 3 Fakultät Für Gesundheitswissenschaften Arbeitsgruppe 3 Epidemiologie & International Public Health, Universität Bielefeld, Bielefeld, Germany; 4 Alice Salomon Hochschule Berlin, Alice-Salomon-Platz 5, 12627 Berlin, Germany; 5 Klinik Für Gynäkologie, Charité Universitätsklinikum, Campus Virchow-Klinikum, Augustenburger Platz 1, 13353 Berlin, Germany; 6 Klinik Für Gynäkologie und Geburtshilfe, St. Joseph Krankenhaus, Wüsthoffstraße 15, 12101 Berlin, Germany

**Keywords:** Birth experience, Refugee health, Immigrant health, Obstetric care, Maternal health, Migrant questionnaire

## Abstract

**Introduction:**

Migration is a worldwide phenomenon leading to challenges for the health care system. There is conflicting evidence on the effect of migration on satisfaction with obstetric care. Our hypothesis was that satisfaction with obstetric care was lower in immigrant women or women with self-defined (sd) refugee status than in non-immigrant women.

**Method:**

In the Pregnancy and Obstetric Care for Refugees (PROREF)-study, funded by the Germany Research Council (DFG), between June 2020 and April 2022 in three hospitals of tertiary care in Berlin women were interviewed 1–3 days after giving birth with the *Migrant Friendly Maternity Care Questionnaire* (MFMCQ). The interview data was linked to the routine perinatal data of the hospital charts. Descriptive analysis and logistic regression analysis were performed to identify factors influencing satisfaction with obstetric care.

**Results:**

A total of 3420 women could be included in the study, with an overall response rate of 77.5%. Satisfaction measured generally high with over 80% of women being satisfied with obstetric care at all three time points: during pregnancy, during labor and birth and after giving birth. We identified two factors associated with an increased chance of being satisfied with obstetric care: sd refugee status (OR 2.57, 95% Cl 1.48–4.44, p-value 0.0008) and being multipara (OR 1.40, 95% CI 1.18–1.67, *p*-value 0.000).

**Conclusion:**

Efforts on improving birth experience should focus on primipara. The reasons for non-immigrant women being less satisfied with care than women with sd refugee status are not fully understood.

**Supplementary Information:**

The online version contains supplementary material available at https://doi.org/10.1007/s40615-025-02529-z.

## Introduction

Migration is a global phenomenon. According to the *International Organization for Migration* (IOM), 3.6% of the world population, i.e., 281 million people are international migrants. The United Nations High Commissioner for Refugees (UNHCR) estimated 2022 worldwide 108.4 million people who are forced to migrate and 35.3 million people to be refugees according to the Geneva Refugee Convention [36]. In international comparison, Germany is a country with increasing net immigration. Between 2015 and 2016 due to rising numbers of refugees from Syria the number of migrants and direct descendants of migrants in Germany rose by 8.5% [10]. In 2021, 22.3 million people in Germany were migrants or direct descendants of migrants, which comprises 27.2% of Germany’s population [11, 12].

A negative birth experience has consequences for women later in life. A study on 617 women giving birth in Sweden between 1989 and 1992 found that after a negative experience during the first birth women had fewer subsequent children and a longer interval to the second baby (RR 1.7, 95% CI 1.3–2.3) [17]. There is also evidence that hints at higher rates of postnatal depression after a negative birth experience [2]. A negative birth experience can have a negative effect on parents’ relationship satisfaction and mental health [29].

Factors that influence birth experience identified by a Canadian study are type of birth; degree of awareness, relaxation, and control; helpfulness of partner support; and being together with the infant following birth [7]. A Swedish study showed the following influencing factors: (1) factors related to unexpected medical problems, such as emergency operative delivery, induction, augmentation of labor, and infant transfer to neonatal care; (2) factors related to the woman’s social life, such as unwanted pregnancy and lack of support from partner; (3) factors related to the woman’s feelings during labor, such as pain and lack of control; and (4) factors that may be easier to influence by the caregivers, such as insufficient time allocated to the woman’s own questions at antenatal checkups, lack of support during labor, and administration of obstetric analgesia [37].

The question of the study was: Is satisfaction of care during pregnancy and birth associated with sociodemographic/clinical characteristics (migration status, language skills, household income, education, parity, age)?

Our hypothesis was that satisfaction with obstetric care was lower in women with self-defined (sd) refugee status or immigrant women than in non-immigrant women.

International data show conflicting evidence on the quality of birth experience of immigrant women in high-income countries [1, 19, 32]. Three studies in Australia showed lower satisfaction among immigrant women than women born in Australia [5, 6] [31]. A later review identified the following reasons for lower satisfaction among immigrant women: problems with communication, difficulties navigating the health system and discrimination [32].

A more recent study from Denmark conducted between 2019 and 2020 among recent immigrant women showed the opposite: a higher risk for less satisfaction in case of high degree of acculturation and high degree of education [1]. A study in the USA between 2004 and 2006 showed a similar tendency [15]. Also, in a study in Sweden regarding postpartum care, women born outside of Sweden were more often satisfied with the care they received compared to women born in Sweden [20]. A study in Brussels between 2019 and 2020 revealed four independent risk factors for a negative satisfaction with care: older age, longer residence in Belgium (> 16 years), fluent knowledge of one of the national languages of Belgium, and the delivery hospital [28].

## Material and Methods

### Data Collection

The Pregnancy and Obstetric Care for Refugees (PROREF) study (subproject of the research group PH-LENS, funded by the German Research Foundation, funding labels DA 1199/5–1 and BO 3384/2–1) had the aim to examine the situation of obstetric care for women, who had to flee their home country, compared to other immigrant women and non-immigrant women. The women were grouped in either sd refugee status, immigrant or non-immigrant. The migration categories were not defined by legal residence status, but by self-information of the respective woman, if she had left her home country fleeing due to famine, war or persecution. As the effect of recent flight was to be assessed, only women who migrated in the summer of 2015 or later were categorized in the sd refugee status group.

Between June 2020 and April 2022, data collection was performed in three hospitals of tertiary care in Berlin/Germany. A modified and shortened version of the Migrant Friendly Maternity Care Questionnaire (MFMCQ) [16] was used to assess migration specific data and the routine perinatal data from the hospital charts was afterwards linked to the migration specific data. For our first study using the MFMCQ in Germany a random number estimation was performed based on the study of Brown and Lumley [6]. Regarding a high difference among groups, 173 women had to be included to achieve a significance level of 0.05 according to the random number estimation (for details, please see [19]). In the subsequent study, we found generally high degrees of satisfaction, so that discrimination among groups was difficult [19]. For the PROREF study, thus, a sevenfold bigger cohort for the two groups immigrant vs non-immigrant was planned. We performed a post hoc power analysis, showing a power of 100% comparing women of sd refugee status with non-immigrant women and a power of 53.7% comparing immigrant women to non-immigrant women (see Table S1).

The questionnaire was provided in English, French, and Arabic by the developing team of authors [26]. For the usage in Germany, the MFMCQ had been translated and adapted into German and Turkish in an earlier study of our study group [19]. For the PROREF study, a questionnaire version in Persian, Kurdish, Russian, and Vietnamese was created.

Data collection was performed 1 to 3 days postpartum on the postpartum ward of the respective participating hospital by a team of female trained interviewers. The members of the interview team spoke German, English, French, Russian, and/or Arabic, and were trained in interview administration before data collection. Women speaking the languages Turkish, Persian, Kurdish, or Vietnamese were contacted together with an in-person or telephone translator. All women of 18 years of age or older, who had given birth to one or more living babies in the delivery room of the respective participating hospital and speaking one of the 9 “study languages,” could be included. Women who were in care of a caseload midwife were not included in the study. Care by a caseload midwife leads to more continuity of care and this per se is associated with greater satisfaction, hence the exclusion in this study [9, 14, 27].

The interviewers contacted the women on the postpartum ward of the study sites daily. Due to personnel shortages, randomly days had to be omitted for data collection. Omitted days could be any day of the week, including weekend days, but generally data collection was also performed on weekends. All women meeting the study criteria on a given day of data collection were approached for study inclusion. Thus, the dates of the data collection were random, but on the dates of data collection we tried to include a representative sample. The women were informed about the study’s goals and modalities and after informed consent the interview was performed immediately afterwards. Subsequently, the perinatal data from the hospital charts of the respective woman was linked to the migration specific data from the MFMCQ in the study data base. The study was earlier described in detail by Seidel and Teschemacher et al. [30].

### Data Analysis

Continuous variables were reported as mean or as median. Categorial variables were expressed as the number of participants and corresponding percentage. After descriptive analysis, differences in characteristics were analyzed using *χ*^2^ tests. The level of significance was defined as *p* < 0.05. Confounders were identified based on published evidence.

Primary outcome measure was satisfaction with care at three time points of obstetric care ((1) during pregnancy, (2) during labor and birth and (3) after birth) dichotomized in (a) very satisfied and satisfied and (b) indifferent, unsatisfied, and very unsatisfied. We also assessed overall satisfaction with care in a satisfaction score encompassing all time points of interest. In the questionnaire, the women could choose between five answers to the question about satisfaction. For the satisfaction score, “very satisfied” was rated with 1 point, “satisfied” with 2 points, “indifferent” with 3 points, “unsatisfied” with 4 points, and “very unsatisfied” with 5 points. The points at all three time points of obstetric care were summed up and divided by three.

We controlled for maternal education, maternal German language skills, maternal age, transfer of the baby to the neonatal intensive care unit (NICU), primipara vs at least second birth (multipara), and mode of birth. Maternal education was grouped in (a) no or any education up to graduation from secondary school, (b) high school diploma, and (c) university education. Maternal age was grouped into four groups (18–24 years, 25–29 years, 30–34 years, and 35–51 years). Transfer of the neonate to the NICU, primipara/multipara and maternal German language skills were dichotomized. We subsumed the self-rated German language skills of women who answered to the respective question “mother tongue,” “very good,” or “good” as “good” and if women answered “sufficient,” “rather bad,” or “none,” we categorized their language skills as “not good.”

Mode of birth was grouped into (a) vaginal birth including spontaneous vaginal delivery and vacuum or forceps extraction, (b) c-section including primary or secondary, or (c) emergency c-section. All data analysis was performed using SAS 9.4.

The ethics committee of the Charité—Universitätsmedizin Berlin approved the PROREF study [EA1/316/1]. The study team followed the guidelines by the Charité for good clinical practice and the requirements of the Berlin data protection act.

## Results

### Study Cohort

Out of a total of 18,636 births during the study period at the three study sites, 17,555 fulfilled the inclusion criteria. Randomly 25% of these (4413 women) were invited to participate in the study. With 3420 women participating in the study, we had a response rate of 77.5% (for further details of the study cohort, see also [30]).

Berlin is the capital of Germany, with 3.76 million inhabitants. The second biggest city in Germany is Hamburg with its 1.88 million inhabitants only approximately half the size of Berlin. Overall, in Germany, 19.2% of inhabitants are not born in Germany. In Berlin, 25.9% of inhabitants have immigrated to Germany within their own lifetime [11, 12]. All three study sites are located in Berlin and centers of tertiary care with more than 1500 births in each study site.

Women with self-defined (sd) refugee status were generally younger and had more often no or a low formal education than immigrant and non-immigrant women. Regarding self-rated German language skills women with sd refugee status had lower rates of proficiency of German than immigrant women. While more than 50% of immigrant women had good or very good German skills in reading, speaking or understanding, more than two thirds of women with sd refugee status had a low proficiency in the German language in all three qualities (see Table 1).

**Table 1:** Sociodemographic characteristics of the study cohort

*N* = 3076 with complete data set			
	**sd refugee status (a)**	**Immigrant (b)**	**Non-immigrant (c)**
**Age**			
18–24 years	54 (29.8%)	78 (6.4%)	142 (8.4%)
25–29 years	47 (26.0%)	236 (19.5%)	320 (19.0%)
30–34 years	49 (27.1%)	474 (39.1%)	626 (37.2%)
35–51 years	31 (17.1%)	425 (35.0%)	594 (35.3%)
Chi^2^	(a vs. c) *p* < 0.0001	(b vs. c) *p* = 0.2142	
Age: mean (Std.)	28.7 (5.9)	32.7 (5.2)	32.4 (5.3)
**Education**			
Secondary education or less	81 (44.8%)	210 (17.3%)	500 (29.7%)
High school diploma	50 (27.6%)	203 (16.7%)	339 (20.2%)
University education	50 (27.6%)	800 (66.0%)	843 (50.1%)
Chi^2^	(a vs. c) *p* < 0.0001	(b vs. c) *p* < 0.0001	
**Reading German**			
Good	58 (32.4%)	715 (61.3%)	
Not good	121 (67.6%)	451 (38.7%)	
Missing: 49			
Chi^2^	(a vs. b) *p* < 0.0001		
**Speaking German**			
Good	37 (20.9%)	687 (56.6%)	
Not good	140 (79.1%)	526 (43.4%)	
Missing: 4			
Chi^2^	(a vs. b) *p* < 0.0001		
**Understanding German**			
Good	56 (31.5%)	770 (66.1%)	
Not good	122 (68.5%)	395 (33.9%)	
Missing: 51			
Chi^2^	(a vs. b) *p* < 0.0001		
**Parity**			
Primipara	73 (40.3%)	667 (55.0%)	968 (57.6%)
Multipara	108 (59.7%)	546 (45.0%)	714 (42.4%)
Chi^2^	(a vs. c) *p* < 0.0001	(b vs. c) *p* = 0.1700	

*sd refugee status*, self-defined refugee status; *std*, standard deviation.

### Satisfaction with Care

Generally, there was a high level of satisfaction (> 80% at all time points) with obstetric care among all participating women. Regarding pregnancy care and postpartum care women with sd refugee status were significantly more satisfied with the care they received. Women with sd refugee status and immigrant women were both more satisfied with care received during pregnancy than non-immigrant women. Satisfied with pregnancy care were 95.0% of women with sd refugee status (*p* < 0.0001) and 85.2% of immigrant women (*p* = 0.0377) as opposed to 82.3% of non-immigrant women.

After birth, women with sd refugee status were especially satisfied with the care they received. Ninety-five percent of women with sd refugee status were satisfied with care (*p* = 0.0009) as opposed to 88.9% of immigrant and 87.1% of non-immigrant women who were respectively satisfied with care.

We found a very high rate of satisfaction (> 94%) with care during labor and birth among all three groups of women. There was no difference regarding satisfaction with care during labor and birth depending on migration status (Table 2).

**Table 2:** Satisfaction with care received at three time points of obstetric care

*N* = 1682 with complete data			
	**sd refugee status (a)**	**Immigrant (b)**	**Non-immigrant (c)**
**Satisfaction with care during pregnancy**			
(very) satisfied	172 (95.0%)	1034 (85.2%)	1385 (82.3%)
Indifferent, (very) unsatisfied	9 (5.0%)	179 (14.8%)	297 (17.7%)
Chi^2^	**(a vs. c) *p* < 0.0001**	**(b vs. c) *p* = 0.0377**	
**Satisfaction with care during labor and birth**			
(very) satisfied	173 (95.6%)	1148 (94.6%)	1582 (94.1%)
Indifferent, (very) unsatisfied	8 (4.47%)	65 (5.4%)	100 (6.0%)
Chi^2^	(a vs. c) *p* = 0.4040	(b vs. c) *p* = 0.5017	
**Satisfaction with care after birth**			
(very) satisfied	173 (95.6%)	1078 (88.9%)	1465 (87.1%)
Indifferent, (very) unsatisfied	8 (4.4%)	135 (11.1%)	217 (12.9%)
Chi^2^	**(a vs. c) *p* = 0.0009**	(b vs. c) *p* = 0.1051	

(1) During pregnancy, (2) during labor and birth and (3) after birth.

*sd refugee status*, self-defined refugee status.

When comparing the computed overall satisfaction score, women with sd refugee status were significantly more satisfied at all three time points of care (*p* = 0.0040, see Table 3). While 87.3% (*n* = 158) women with sd refugee status were satisfied with maternity care, only 73.2% of immigrant women (*n* = 888) and 69.7% (*n* = 1172) of non-immigrant women were satisfied.

**Table 3:** Overall satisfaction with obstetric care

	*N*	Mean/Std	Min	Max	*p*
sd refugee status (a)	181	1.52/0.52	1.00	3.33	**0.0040**
Immigrant (b)	1213	1.62/0.53	1.00	3.33	0.2137
Non-immigrant (c)	1682	1.64/0.55	1.00	3.67	

*sd refugee status*, self-defined refugee status; *std*, standard deviation.

Among women who have low German language proficiency, there is a trend to higher chance for satisfaction with maternity care, adjusted for age, and degree of education. Women who self-rated their German understanding as not good have a higher satisfaction with obstetric care than women with good German understanding (*p* = 0.0493). The *p*-value does not reach the level of significance for the self-rated German language skills in reading and speaking though (see Table 4).

**Table 4:** Satisfaction with obstetric care depending on self-rated German language skills

	Odds ratio	95% confidence interval	*p*-value
**German reading**			
Good	Reference		
Not good	1.23	0.95–1.59	0.1118
**German speaking**			
Good	Reference		
Not good	1.22	0.95–1.56	0.1162
**German understanding**			
Good	Reference		
Not good	1.30	1.00–1.69	0.0493

All models adjusted for maternal age and education.

Controlling for confounders maternal education, maternal age NICU transfer of the neonate and mode of birth did not have an effect on satisfaction with care. Migration status remained significant, with an OR of 3.02 of being satisfied with care with sd refugee status. Furthermore, we found women giving birth the first time in their life had a lower chance of satisfaction with care than women who had given birth to at least one child earlier in their life (see Table 5).

**Table 5:** Chance of being satisfied with obstetric care

*N* = 2981, events = 2141			
	OR	95% KI	*p*-Wert
**Migration status**			
sd refugee status	3.02	1.89–4.83	** < 0.0001**
Immigrant	1.14	0.96–1.35	0.1417
Non-immigrant	1.00		
**Maternal education**			
Secondary education or less	0.91	0.73–1.13	0.3700
High school diploma	0.96	0.77–1.20	0.7020
University education	1.00		
**Maternal age**			
18–24 years	1.00		
25–29 years	0.98	0.71–1.37	0.9224
30–34 years	1.15	0.84–1.59	0.3845
35–51 years	1.21	0.86–1.69	0.2715
**NICU transfer**			
No	1.00		
Yes	1.02	0.73–1.44	0.9065
**Parity**			
Primipara	1.00		
Multipara	1.41	1.19–1.67	** < 0.0001**
**Mode of birth**			
Vaginal birth	1.00		
c-section (primary or secondary)	0.95	0.81–1.13	0.5744
Emergency c-section	0.76	0.37–1.55	0.4523

Logistic regression analysis model 1: including confounders: maternal education, maternal age, NICU transfer of neonate, primipara vs. multipara and mode of birth.

*CI*, confidence interval; *NICU*, neonatal intensive care unit; *OR*, odds ratio; *sd refugee status*, self-defined refugee status.

Event = satisfied with care during pregnancy, during labor and birth and after birth.

Adding German language skills as a confounder to the logistic regression model did not change the overall results. Again, women with sd refugee status and women giving birth to at least their second child had a higher chance of being satisfied with care (see Table 6). When German was not the native language, there was a trend to more satisfaction with care, although this again did not reach a significant *p*-value.

**Table 6:** Chance of being satisfied with obstetric care

*N* = 2981, events = 2141			
	OR	95% CI	*p*-Wert
**Migration status**			
sd refugee status	2.57	1.48–4.44	**0.0008**
Immigrant	1.00	0.74–1.34	0.9809
Non-immigrant	1.00		
**Maternal education**			
Secondary education or less	0.91	0.73–1.13	0.3760
High school diploma	0.96	0.77–1.20	0.7007
University education	1.00		
**Maternal age**			
18–24 years	1.00		
25–29 years	0.99	0.71–1.37	0.9268
30–34 years	1.16	0.84–1.61	0.3547
35–51 years	1.22	0.87–1.71	0.2393
**NICU transfer**			
No	1.00		
Yes	1.03	0.73–1.44	0.8827
**Number of previous birth**			
Primipara	1.00		
Multipara	1.40	1.18–1.67	**0.0001**
**Mode of birth**			
Vaginal birth	1.00		
c-section (primary or secondary)	0.96	0.81–1.13	0.6119
Emergency c-section	0.76	0.37–1.55	0.4501
Maternal German language proficiency*			
Native language	1.00		
Good	1.13	0.84–1.52	0.4059
Not good	1.23	0.86–1.75	0.2582

Logistic regression analysis model 2: model 1 (see Table 5) expanded by maternal German language skills*. Counfounders: maternal education, maternal age, NICU transfer of neonate, primipara vs. multipara and mode of birth, maternal German language skills*

*CI*, confidence interval; *NICU*, neonatal intensive care unit; *OR*, odds ratio; *sd refugee status*, self-defined refugee status.

Event = satisfied with care during pregnancy, during labor and birth and after birth.

*Index composed of German_understanding, German_speaking, and German_reading.

## Discussion

The hypothesis of our study, expecting immigrant women to be less satisfied with the obstetric care they received, proved negative. In our study, we identified being multipara, sd refugee, or immigrant as factors leading to more satisfaction with obstetric care. There is conflicting evidence on the satisfaction of immigrant women in high-income countries. In the *Survey of Recent Mothers* conducted in 1989 in Australia, immigrant women from non-English-speaking countries were more often unsatisfied (28.3%) with prenatal care than women born in Australia (12.3%). In this postal survey, the brief explanation of the study was available in six community languages, but the questionnaire was only provided in English. The authors identify this as the reason, why non-English-speaking women were underrepresented in the study sample [5]. A follow-up study in 1993 showed a similar picture: immigrant women from non-English-speaking countries were less satisfied with the care received. Only 28.5% of immigrant women from non-English-speaking countries rated their pregnancy care as “very good,” while non-immigrant women did so in 73.3% [6]. In this study, immigrant women with low English proficiency were most probably again underrepresented as the cover letter inviting women to participate was translated into six other languages than English, but the questionnaire itself was not provided in other languages than English. In a parallel study on women from Vietnam, Turkey and the Philippines conducted to learn more about the satisfaction with pregnancy care of immigrant women; the satisfaction with care was dependent on the English skills of the women. Women with poor English proficiency were only in 15.1% fully satisfied with pregnancy care, while women with good English proficiency were fully satisfied with their pregnancy care in 41.4%. This study showed the same trend regarding care during labor and birth. Fully satisfied were 31.8% of immigrant women with poor English proficiency vs. 57.6% with good English proficiency. After delivery, immigrant women speaking English well were satisfied with care in 42.4% while the ones speaking English not well were only satisfied with postpartum care in 26.0%. Other reasons for dissatisfaction with care besides a language barrier were feeling less welcomed, not being involved in decisions and being treated with prejudice by the health staff [31]. According to a review on studies from five high-income-countries, communication problems, problems navigating the health system, and discrimination are the key factors leading to less satisfaction with care among immigrant women [32].

Our study results differ from the data from Australia. Generally, we had a very high rate of satisfaction with obstetric care. We found higher satisfaction with pregnancy care among immigrant women and women with sd refugee status compared to non-immigrant women. There was no difference in satisfaction depending on the migration status regarding satisfaction with care during labor and birth. Regarding satisfaction with care after delivery again, women with sd refugee status were the most satisfied group. Looking at the language skills as a factor, there was a trend to more satisfaction when the German language proficiency was low. In a former study in a smaller study cohort conducted in 2017, we found the same trend to more satisfaction with lower German proficiency [19]. In a study from Denmark conducted between 2019 and 2020, recent immigrant women (living in Denmark less than 5 years) were asked with the MFMCQ about their satisfaction with obstetric care. Risk factors for being less satisfied were having very good Norwegian language skills, having a Norwegian partner and having a higher degree of education [1]. A study on 1243 women in the USA between 2004 and 2006 showed less satisfaction with obstetric care among more acculturated women [15]. A study evaluating postpartum care showed higher satisfaction regarding medical and emotional aspects of the care received among women born outside of Sweden compared to women born in Sweden [20]. Schönborn et al. identified longer residence in Belgium and fluency of one of the national languages of Belgium as risk factors for less satisfaction with received obstetric care in Brussels [28]. Thus, there seems to be a tendency to less satisfaction among more acculturated immigrant women.

More satisfaction with care among less acculturated immigrant women might be explained through different expectations of care, as Bains et al. claimed in the discussion of their results from Norway [1]. Furthermore, more recently immigrated women from low-income countries might compare the care they received in their new country of residence to the medical standard in their country of origin and be grateful for the higher standard of care. Medical staff interviewed in a qualitative study in Berlin, voiced this gratefulness for high medical standards as an explanation for higher satisfaction with care among immigrant women [18]. The MFMCQ is recommended to be administered as a phone interview several weeks after the birth. The mentioned studies in Australia were conducted with written questionnaires sent to the women weeks after giving born [4, 6]. In our study, in the Belgian study and the study in Norway by Bain et al., the data was collected within days after birth. There is data suggesting that immediately after birth of a healthy child women are so relieved that they regard the past more positive than weeks later [3]. Furthermore, friendly contact with the multilingual interviewing study members might have had a positive effect in the time of the COVID-19 pandemic, when visitors were generally not allowed in the hospitals. The data from Sweden, showing more satisfaction among immigrant women with several aspects of postpartum care was conducted as a postal questionnaire several weeks after birth [20]. Another methodological problem could be that immigrants are known to have different response strategies to cross-cultural surveys. Morren et al. in their paper from 2014 define “behavioral response strategy,” “attitude-detached response strategy,” and “attitude-balanced response strategy” [24]. The “attitude-detached response strategy” is characterized by a generalization detached from the personal feelings to comply with a supposed majority opinion. We cannot exclude that immigrant and women with sd refugee status in our cohort did answer more with a “attitude-detached response strategy.”

Even if women with sd refugee status and immigrant women voiced high degrees of satisfaction with care in our study, a review of qualitative studies from immigrant women to European countries revealed that immigrant women want to be treated without prejudices [13]. We speculate that speaking better German might lead to higher awareness for being treated with prejudices, thus leading to the trend of less satisfaction among immigrant and women with sd refugee status with better German proficiency. This hypothesis needs to be tested in further studies, preferably with ethnographic methods.

Some of the participating immigrant women or women with sd refugee status mentioned in the interviews that during labor and birth they were treated in their native language. The increasing employment of immigrants in the health care sector in Germany, with approximately 12.5% of doctors from abroad and 7.9% of nurses with a migration background [8] could be a confounding factor. We did not evaluate in our study if satisfaction with care for immigrant and women with sd refugee study was higher because they received care in their native language or support by a qualified interpreter. Additionally, the interviewing study team spoke various languages and sometimes helped in the clinical routines when data collection and problems in the clinical routine coincided. This sporadic help might have led to increased satisfaction among women with low German proficiency. Data on this sporadic help through the study team was not systematically gathered and thus could not be included in the analysis as a confounding variable.

In their review, Fair et al. discuss that pregnant immigrant women on top of the stress all women have when being pregnant and having a newborn are additionally stressed by factors associated with their migration. Fair et al. highlight the extra burden on immigrant women by navigating the new health care system, their struggle with socioeconomic problems and potential mental health issues by traumatic experiences before or during migration [13]. These aspects should not be forgotten when delivering care to immigrant and refugee women even if they voice high degrees of satisfaction.

Our study hypothesis that immigrant women are at increased risk for dissatisfaction proved negative. On the contrary, we identified non-immigrant primipara at being at increased risk for dissatisfaction. It remains unclear why non-immigrant women are least satisfied with care during pregnancy and less satisfied than women with sd refugee status after birth. Again, here different expectations could lead to less satisfaction. A Canadian study showed, for example, that attendance of prenatal-classes led to lower satisfaction with the birth experience [33]. In an interview study conducted among staff in delivery rooms in Berlin, midwives and doctors suppose that a “natural take on giving birth” is leading to more satisfaction with birth among immigrant women [18]. Thus, non-immigrant women might have too many expectations on their birth experience. A study on the effect of a written birth plan on a positive birth experience among 302 women in the USA showed a high rate of satisfaction with birth when more wishes in the birth plan were fulfilled (*p* = 0.03), but satisfaction decreased by 80% if there was a higher number of requests for the respective birth (*p* < 0.01) [23].

Our results are remarkable in another aspect. The study was conducted during the COVID-19 pandemic. There is evidence that migrants and refugees worldwide in general showed higher levels of increased discrimination and increases in daily life stressors, especially women [35]. In a cohort study in Italy on Italian women with a historic control group, the satisfaction of women was not altered during the pandemic [21]. A study in the USA identified Black and Latina women as a group at increased risk for discrimination during pregnancy and subsequent less satisfaction with birth experience [22]. Data in Germany on women giving birth during the pandemic showed similar levels of satisfaction with birth experience, while support during birth was perceived to be higher under the pandemic restrictions. The participating German mothers though expressed significantly higher dissatisfaction with the maternal role during the pandemic [25].

Different to the situation in the USA the problem of discrimination seems to be not so relevant regarding satisfaction with care for immigrant and women with sd refugee status. The unexpected lower satisfaction of non-immigrant women might be explained by unmet expectations. On the other hand, women with sd refugee status often lived outside of the hospital in reception centers. Especially for women living in reception centers, the hospital might have been a very positive improvement in terms of facilities and living conditions. While in reception centers, many people share for example the sanitary facilities and privacy is sparse. On the contrary, in the participating hospitals on the postpartum ward at the most two women shared sanitary facilities at the same time.

It is interesting that transfer of the newborn to the NICU did not have an effect on the satisfaction with care of the mother in our study. A study in Canada from October 2004 to December 2005 found less satisfaction after birth when the mother was separated from the infant [7]. A Swedish study from 1999 showed the same result [37]. Potentially now approximately 20 years later, the routines of the NICU have substantially changed. Introduction of kangarooing and more involvement of parents in the care for children on the NICU might make the impact of separation of mother and child feel less.

Some studies show an effect of the mode of birth on satisfaction with birth experience. A study in Sweden highlighted emergency operative delivery as a risk factor for a negative birth experience [37]. On the other hand, a German study did not find an effect of the mode of delivery on satisfaction with birth experience [34].

Our study showed risk factors for not being satisfied with care. Further studies in Germany are needed to investigate the mechanism how primipara are less satisfied with obstetric care. Ethnographic studies can be helpful to facilitate a deeper understanding of different expectations among different ethnic groups and the subsequent effects on satisfaction with care. Discrimination as a potential explaining factor for less satisfaction among well acculturated immigrant women should be studied with qualitative methods. Respective interventions should be designed. [8, 13, 19, 27]

### Strength and Limitations of the Study

- Strength: (1) The PROREF study is the largest prospective quantitative study in Germany on the peripartal health of women with sd refugee status. (2) There was a high response rate of 77.5% of the women invited to participate despite the COVID pandemic. (3) Relevant factors for a positive birth experience were identified. (4) Due to in-person standardized interviews by a multilingual team of female interviewers, the extensive use of language interpreters and the recruitment on the postpartum ward, women usually underrepresented in other studies could be included.
- Limitations: (1) It remains questionable if the study sample can be regarded as representative, as it was only conducted in centers of tertiary care in Germany’s capital Berlin. (2) We do not know if the results of this study also apply to women having recently fled from Ukraine. (3) The study was only conducted in Berlin. The results might not be transferable to rural parts of Germany. (4) There might be a recency bias. Birth experience might be evaluated differently with greater timely distance.

## Conclusions and Recommendations

The overall high satisfaction with obstetric care in Berlin/Germany is a comforting result. Efforts on improving birth experience should focus on primipara.

Women with sd refugee status seem to have lower expectations regarding their birth experience than non-immigrant women. The reasons for non-immigrant women being less satisfied with care than women with sd refugee status are not fully understood. Management of expectations regarding labor and birth could be a useful intervention here.

## Supplementary Information

Below is the link to the electronic supplementary material.

Supplementary file1 (DOCX 14.8 KB)

## Data Availability

More data is available on request.

## References

[1] Bains S, Sundby J, Lindskog BV, Vangen S, Diep LM, Owe KM, Sorbye IK. Satisfaction with maternity care among recent migrants: an interview questionnaire-based study. BMJ Open. 2021;11(7):e048077. 34272220 10.1136/bmjopen-2020-048077 PMC8287626

[2] Bell AF, Andersson E. The birth experience and women’s postnatal depression: a systematic review. Midwifery. 2016;39:112–23. 27321728 10.1016/j.midw.2016.04.014

[3] Britton JR. The assessment of satisfaction with care in the perinatal period. J Psychosom Obstet Gynecol. 2012;33(2):37–44. 10.3109/0167482X.2012.658464 22554136

[4] Brown S, Lumley J. Antenatal care: a case of the inverse care law? Aust N Z J Public Health. 1993;17(2):95–103. 10.1111/j.1753-6405.1993.tb00115.x 8399722

[5] Brown S, Lumley J. Satisfaction with care in labor and birth: a survey of 790 Australian women. Birth. 1994;21(1):4–13. 8155224 10.1111/j.1523-536x.1994.tb00909.x

[6] Brown S, Lumley J. Changing childbirth: lessons from an Australian survey of 1336 women. Br J Obstet Gynaecol. 1998;105(2):143–55. 9501778 10.1111/j.1471-0528.1998.tb10044.x

[7] Bryanton J, Gagnon AJ, Johnston C, Hatem M. Predictors of women’s perceptions of the childbirth experience. Journal of obstetric, gynecologic, and neonatal nursing : JOGNN / NAACOG. 2008;37(1):24–34. 10.1111/j.1552-6909.2007.00203.x 18226154

[8] Can E, Konrad CM, Khan-Gökkaya S, Molwitz I, Nawabi J, Yamamura J, Hamm B, Keller S. Foreign healthcare professionals in Germany: a questionnaire survey evaluating discrimination experiences and equal treatment at two large university hospitals. Healthcare MDPI; 2022. 10.3390/healthcare10122339 PMC9777572 36553863

[9] D’Haenens F, Van Rompaey B, Swinnen E, Dilles T, Beeckman K. The effects of continuity of care on the health of mother and child in the postnatal period: a systematic review. Eur J Public Health. 2019. 10.1093/eurpub/ckz082 31121019

[10] DESTATIS. Statistische Bundesamt: Pressemitteilung Nr. 261 vom 01.08.2017; 2017. https://www.destatis.de/DE/PresseService/Presse/Pressemitteilungen/2017/08/PD17_261_12511.html. Accessed 16 Mar 2018.

[11] DESTATIS. Bevölkerung in Privathaushalten nach Migrationshintergrund und Bundesländern. 2023. https://www.destatis.de/DE/Themen/Gesellschaft-Umwelt/Bevoelkerung/Migration-Integration/Tabellen/migrationshintergrund-laender.html. Accessed 1 Jan 2025.

[12] DESTATIS. Migration und Integration. 2023. https://www.destatis.de/DE/Themen/Gesellschaft-Umwelt/Bevoelkerung/Migration-Integration/_inhalt.html. Accessed 16 Mar 2023.

[13] Fair F, Raben L, Watson H, Vivilaki V, van den Muijsenbergh M, Soltani H, O. team,. Migrant women’s experiences of pregnancy, childbirth and maternity care in European countries: a systematic review. PLoS ONE. 2020;15(2):e0228378. 32045416 10.1371/journal.pone.0228378 PMC7012401

[14] Floris L, Irion O, Bonnet J, Politis Mercier MP, de Labrusse C. Comprehensive maternity support and shared care in Switzerland: comparison of levels of satisfaction. Women Birth. 2018;31(2):124–33. 28711398 10.1016/j.wombi.2017.06.021

[15] Fuentes-Afflick E, Odouli R, Escobar GJ, Stewart AL, Hessol NA. Maternal acculturation and the prenatal care experience. J Women’s Health. 2014;23(8):688–706. 10.1089/jwh.2013.4585 PMC4129971 24979178

[16] Gagnon AJ, DeBruyn R, Essen B, Gissler M, Heaman M, Jeambey Z, Korfker D, McCourt C, Roth C, Zeitlin J, Small R. Development of the Migrant Friendly Maternity Care Questionnaire (MFMCQ) for migrants to Western societies: an international Delphi consensus process. BMC Pregnancy Childbirth. 2014;14:200. 24916892 10.1186/1471-2393-14-200 PMC4088918

[17] Gottvall K, Waldenstrom U. Does a traumatic birth experience have an impact on future reproduction? BJOG. 2002;109(3):254–60. 11950179 10.1111/j.1471-0528.2002.01200.x

[18] Großkreutz C, Gürbüz B, Borde T, Rancourt RC, Henrich W, David M, Seidel V. Equal alternatives or lower standards for immigrant women—analyzing obstetric care for immigrant women in Berlin within the framework of cultural health capital. J Racial Ethn Health Disparities. 2024;11:2689–98. 37581765 10.1007/s40615-023-01732-0 PMC11480289

[19] Gürbüz B, Großkreutz C, Vortel M, Borde T, Stepan H, Rancourt RC, Sauzet O, Henrich W, David M, Seidel V. The influence of migration on women’s satisfaction during pregnancy and birth: results of a comparative prospective study with the Migrant Friendly Maternity Care Questionnaire (MFMCQ). Arch Gynecol Obstet. 2019;300:555–67. 31267197 10.1007/s00404-019-05227-4

[20] Hildingsson I, Parment H, Öhrn U, Johansson M. Foreign-born women rated medical and emotional aspects of postnatal care higher than women born in Sweden: a quantitative comparative study. Eur J Midwifery. 2023;7(11):32–40. 38023945 10.18332/ejm/172573 PMC10644228

[21] Inversetti A, Fumagalli S, Nespoli A, Antolini L, Mussi S, Ferrari D, Locatelli A. Childbirth experience and practice changing during COVID-19 pandemic: a cross-sectional study. Nurs Open. 2021;8(6):3627–34. 34002943 10.1002/nop2.913 PMC8242706

[22] Janevic T, Maru S, Nowlin S, McCarthy K, Bergink V, Stone J, Dias J, Wu S, Howell EA. Pandemic birthing: childbirth satisfaction, perceived health care bias, and postpartum health during the COVID-19 pandemic. Matern Child Health J. 2021;25(6):860–9. 33909205 10.1007/s10995-021-03158-8 PMC8079857

[23] Mei JY, Afshar Y, Gregory KD, Kilpatrick SJ, Esakoff TF. Birth plans: what matters for birth experience satisfaction. Birth. 2016;43(2):144–50. 26915304 10.1111/birt.12226

[24] Morren M, Gelissen JP, Vermunt JK. Response strategies and response styles in cross-cultural surveys. Cross-Cult Res. 2012;46(3):255–79.

[25] Perez A, Schepanski S, Göbel A, Stuhrmann LY, Singer D, Bindt C, Mudra S. Experience of early motherhood during the first wave of the COVID-19 pandemic in Northern Germany: a single-centre before and after comparison. J Reprod Infant Psychol. 2023;41(4):428–44. 34918988 10.1080/02646838.2021.2013458

[26] ROAM. (2014). Additional file 2–4: the MFMCQ in English, French and Spanish Version_17Apr2014. Retrieved accessed on Dec 5 2016, from https://www.ncbi.nlm.nih.gov/pmc/articles/PMC4088918/

[27] Sandall J, Soltani H, Gates S, Shennan A, Devane D. Midwife-led continuity models versus other models of care for childbearing women. Cochrane database of systematic reviews. 2016;(4). 10.1002/14651858.CD004667.pub5 PMC8663203 27121907

[28] Schönborn C, Castetbon K, De Spiegelaere M. Maternal birthplace and experiences of perinatal healthcare in Belgium: evidence from a cross-sectional survey. Midwifery. 2024;138:104139. 39154598 10.1016/j.midw.2024.104139

[29] Seefeld L, Handelzalts JE, Horesh D, Horsch A, Ayers S, Dikmen-Yildiz P, KömürcüAkik B, Garthus-Niegel S. Going through it together: Dyadic associations between parents’ birth experience, relationship satisfaction, and mental health. J Affect Disord. 2024;348:378–88. 38154585 10.1016/j.jad.2023.12.044

[30] Seidel V, Teschemacher L, Breckenkamp J, Henrich W, Borde T, David M, Abou-Dakn M. Geburtsmedizinische Versorgung bei Gestationsdiabetes von geflüchteten und immigrierten Frauen im Vergleich zu nicht-immigrierten Frauen in Berlin: Eine Analyse quantitativer Daten der Pregnancy and Obstetric Care for Refugees (PROREF)-Studie. Z Geburtshilfe Neonatol. 2024;228(03):260–9. 38373724 10.1055/a-2238-3364

[31] Small R, Rice PL, Yelland J, Lumley J. Mothers in a new country: the role of culture and communication in Vietnamese, Turkish and Filipino women’s experiences of giving birth in Australia. Women Health. 1999;28(3):77–101. 10374809 10.1300/J013v28n03_06

[32] Small R, Roth C, Raval M, Shafiei T, Korfker D, Heaman M, McCourt C, Gagnon A. Immigrant and non-immigrant women’s experiences of maternity care: a systematic and comparative review of studies in five countries. BMC Pregnancy Childbirth. 2014;14:152. 24773762 10.1186/1471-2393-14-152 PMC4108006

[33] Smarandache A, Kim TH, Bohr Y, Tamim H. Predictors of a negative labour and birth experience based on a national survey of Canadian women. BMC Pregnancy Childbirth. 2016;16(1):114. 27193995 10.1186/s12884-016-0903-2 PMC4870779

[34] Spaich S, Welzel G, Berlit S, Temerinac D, Tuschy B, Sütterlin M, Kehl S. Mode of delivery and its influence on women’s satisfaction with childbirth. Eur J Obstet Gynecol Reprod Biol. 2013;170(2):401–6. 23962715 10.1016/j.ejogrb.2013.07.040

[35] Spiritus-Beerden E, Verelst A, Devlieger I, Langer Primdahl N, BotelhoGuedes F, Chiarenza A, De Maesschalck S, Durbeej N, Garrido R, Gaspar de Matos M. Mental health of refugees and migrants during the COVID-19 pandemic: the role of experienced discrimination and daily stressors. Int J Environ Res Public Health. 2021;18(12):6354. 34208243 10.3390/ijerph18126354 PMC8296172

[36] UNHCR (2022). Global trends. https://www.unhcr.org/global-trends-report-2022#:~:text=At%20the%20end%20of%202022,events%20seriously%20disturbing%20public%20order. accessed on 1.2.2024.

[37] Waldenstrom U, Hildingsson I, Rubertsson C, Radestad I. A negative birth experience: prevalence and risk factors in a national sample. Birth. 2004;31(1):17–27. 15015989 10.1111/j.0730-7659.2004.0270.x

