# Supplementary material for: Satisfaction with Maternity Care Among Immigrant Women, Refugee Women and Non-immigrant Women: Results of the Pregnancy and Obstetric Care for Refugees (PROREF) Cross-Sectional Study
Source: J Racial Ethn Health Disparities. 2025 Jul 2;13(5):3713–22. doi: 10.1007/s40615-025-02529-z (PMC13546336; doi:10.1007/s40615-025-02529-z)
Supplement: Supplementary file 1 [file 40615_2025_2529_MOESM1_ESM.docx]

| **Table S1: Post-hoc power analysis** | | | |
| --- | --- | --- | --- |
| **Overall Satisfaction Score: sd refugee status vs. non-immigrants**  sd refugee status: self-defined refugee status | | **Overall Satisfaction Score: immigrant women vs. non-immigrant women** | |
| **sd refugee status** | **87.3 %** | **Immigrant** | **73.2 %** |
| **Non-immigrant** | **69.7 %** | **Non-immigrant** | **69.7 %** |
| **sd refugee status N** | **181** | **Immigrant          N** | **1213** |
| **Non-immigrant N** | **1682** | **Non-immigrant N** | **1682** |
| **Alpha** | **0.05** | **Alpha** | **0.05** |
| Power | 100.0 % | Power | 53.7 % |
